# Attack Resilience of the Evolving Scientific Collaboration Network

**DOI:** 10.1371/journal.pone.0026271

**Published:** 2011-10-14

**Authors:** Xiao Fan Liu, Xiao-Ke Xu, Michael Small, Chi K. Tse

**Affiliations:** 1 Department of Electronic and Information Engineering, The Hong Kong Polytechnic University, Hung Hom, Kowloon, Hong Kong; 2 School of Communication and Electronic Engineering, Qingdao Technological University, Qingdao, People's Republic of China; Umeå University, Sweden

## Abstract

Stationary complex networks have been extensively studied in the last ten years. However, many natural systems are known to be continuously evolving at the local (“microscopic”) level. Understanding the response to targeted attacks of an evolving network may shed light on both how to design robust systems and finding effective attack strategies. In this paper we study empirically the response to targeted attacks of the scientific collaboration networks. First we show that scientific collaboration network is a complex system which evolves intensively at the local level – fewer than 20% of scientific collaborations last more than one year. Then, we investigate the impact of the sudden death of eminent scientists on the evolution of the collaboration networks of their former collaborators. We observe in particular that the sudden death, which is equivalent to the removal of the center of the egocentric network of the eminent scientist, does not affect the topological evolution of the residual network. Nonetheless, removal of the eminent hub node is exactly the strategy one would adopt for an effective targeted attack on a stationary network. Hence, we use this evolving collaboration network as an experimental model for attack on an evolving complex network. We find that such attacks are ineffectual, and infer that the scientific collaboration network is the trace of knowledge propagation on a larger underlying social network. The redundancy of the underlying structure in fact acts as a protection mechanism against such network attacks.

## Introduction

Many natural and man-made complex systems such as biological networks, the WWW, airport network and stock markets network, evolve intensively at the local level [1]–[3]. In fact, local level evolution is both characteristic and typical of human dynamics, where people constantly change their affinity, cooperation strategies and communication patterns [4]–[6]. There are now several notable models of network evolution including the preferential attachment model [7] and the adaptive network models in which network topology evolves as a feedback to the state change of nodes [8]. However in the real world, both network nodes and edges may appear and disappear through a variety of other mechanisms. For example, none of the above mentioned models consider the life span of connections among nodes, which may naturally have a broad distribution uncorrelated with their topological properties.

On the other hand, it has been widely observed that many stationary networks are robust to random failure but vulnerable to targeted attacks [9], [10]. For example, the analysis of the North America blackout in 2003 shows that disturbances affecting key transmission substations greatly reduce the grid's ability to function [11]. Immunization strategies based on the network vulnerability have also been proposed to stop epidemic spreading on complex networks [12]. The scientific collaboration network, which bears the same statistical properties as many stationary complex networks [13], has also been shown, in numerical simulations, to be vulnerable to targeted removal of important nodes [9]. However, exactly how the intensively evolving scientific collaboration network responds to such attacks in the real world has not been carefully studied.

In this paper we analyze the collaboration network of US-based life scientists to address two main topics. First, we examine the topological evolution of the network and show that the scientific collaboration network is intensively evolving. When compared to recently proposed theoretical models of such networks [14]–[18] we find that the data is consistent with changes in link configuration being driven by an autonomous process, rather than in response to the change of state of adjacent nodes. Second, we analyze the impact of unanticipated death of high profile scientists to their collaborators' collaboration network building. We use sudden death within the network as an observed experimental proxy for targeted attack on an evolving complex network. We find the network to be very robust against the removal of even these hub nodes. Furthermore, we conjecture that the scientific collaboration network should be considered the trace of knowledge spreading on a larger and denser mapping of hidden social ties among scientists. That is, not only is there a network of active collaboration, but there is a secondary larger hidden network of latent potential collaborations. When nodes in the active collaboration network are removed, this latent network helps to replace that structure in a robust manner.

## Results

### Topological evolution on the networks

Collaborations between scientists do not last forever. In the scientific collaboration network – where nodes are scientists and links are collaborations – the network can therefore have drastically different constitution when sampled in different time intervals. In this section we study the topological evolution on the collaboration network first by examining the life span of scientific collaborations. Five thousand scientists are sampled from the AAMC Faculty Roster according to the criteria that their academic life spans are longer than 10 years and each of them has more than 10 collaborators. By using this criterion, we actually assure that the life span of collaboration will not be restricted by the observation period. Then by retrieving their publications from PubMed, the life span and productivity (in term of numbers of journal articles published) of each pair of collaboration can be studied.

**Figure 1:**
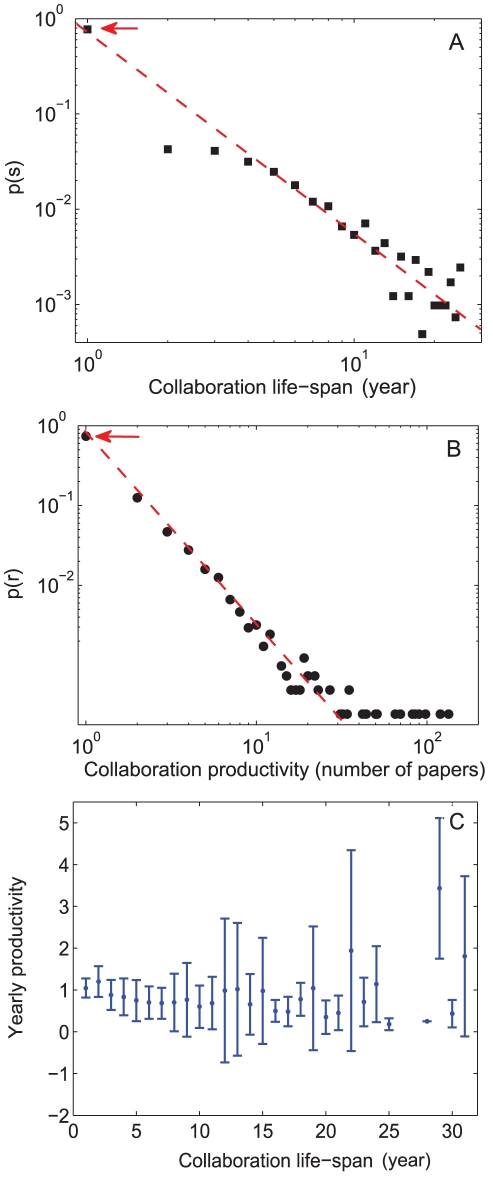
Statistics of scientific collaboration life span and productivity. (A) The probability distribution *p*(*s*) of collaboration life span *s*. Most collaborations lasts for only one year. The dashed line is a power law with exponent −2.3. (B) The probability distribution *p*(*r*) of number of journal articles *r* published by each collaboration. The dashed line is a power law with exponent −2.4. (C) Average yearly productivity for collaboration of different life spans. The average publications produced by each pair in a collaboration stays a bit lower than 1 per year, whereas for collaborations with longer life spans, the productivity varies.

**Figure 2:**
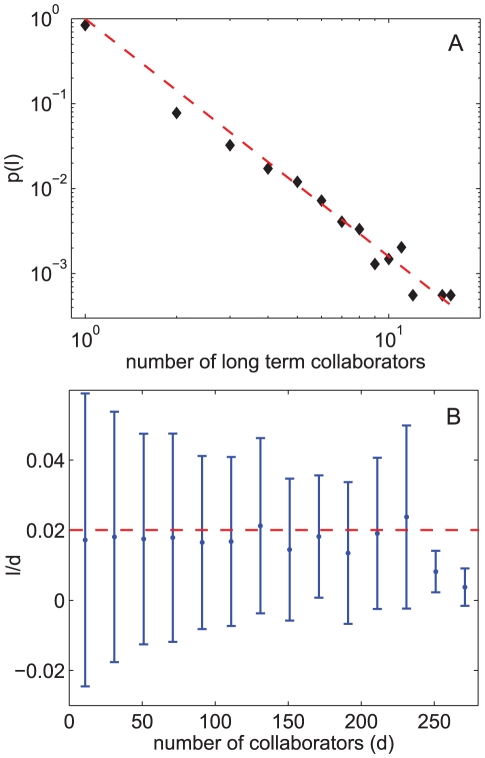
Statistics of long term scientific collaborations. (A) The probability distribution *p*(*l*) of number of long term collaborations 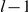 a scientist can have. The average number of long term collaborations the 5000 selected scientists have is 0.73. Most of the scientists do not have long term collaborations with peer researchers. The dashed line is a power law with exponent −2.4. (B) The correlation between the a scientist's number of collaborators *d* and probability of having long term collaborators (denoted by the proportion of long term ones in all the collaborators 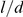). Each point on the graph shows the average 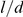 ratio of scientists with 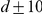 collaborators.

Figs. 1A and B show the distribution of life span *s* and the distribution of collaboration productivity *r* of all pairs of scientific collaborations. The extremely skewed distributions indicate that most of the scientific collaborations last for only one year and have only one research article published. Fig. 1C shows the correlation between scientific collaboration life span and output. There is no clear evidence that collaborations with long life span will have higher productivity than those with short life span. Notice from Fig. 1A that fewer than 20% of scientific collaboration last more than one year, indicating that long term collaborations are actually fairly infrequent. We define the “long term” collaborations of a researcher as those last longer than half of the scientist's academic life span. As shown in Fig. 2A, the probability *P*(*l*) of a scientist having *l*−1 long term collaborators roughly decays with a power law, where most of the scientists have no long term collaborators. Fig. 2B shows that the number of long term collaborators to total number of collaborators ratio 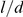 stays stationary for all degrees, which means that no matter how big the collaboration network a scientist has, approximately among every 50 of his/her collaborators, only one will turn out to be a long term collaboration. Hence, the dominance of short life span collaborations characterizes the scientific collaboration network as an intensively evolving network. The lack of competitive advantage of long term collaborations to short term ones in both productivity and team building actually implies that when selecting collaborators, short term collaborations are intrinsically preferred over long term long term co-operation.

To fully characterize the dynamics of the topological network evolution, egocentric scientific collaboration networks are constructed based on a sliding window. The egocentric network of a scientist contains the scientist and his/her first tier collaborators, i.e. the scientists co-authored papers with him/her, and/or the second tier collaborators, i.e. the co-authors of the first tier collaborators excluding the center scientist him/her-self, within a certain period of time. Here we consider the egocentric networks in two different scales:

1. T-1 network: (i) the center node (the scientist) and (ii) its first tier neighbors;
2. T-2 network: (i) the center node (the scientist), (ii) its first tier neighbors and (iii) second tier neighbors.

**Figure 3:**
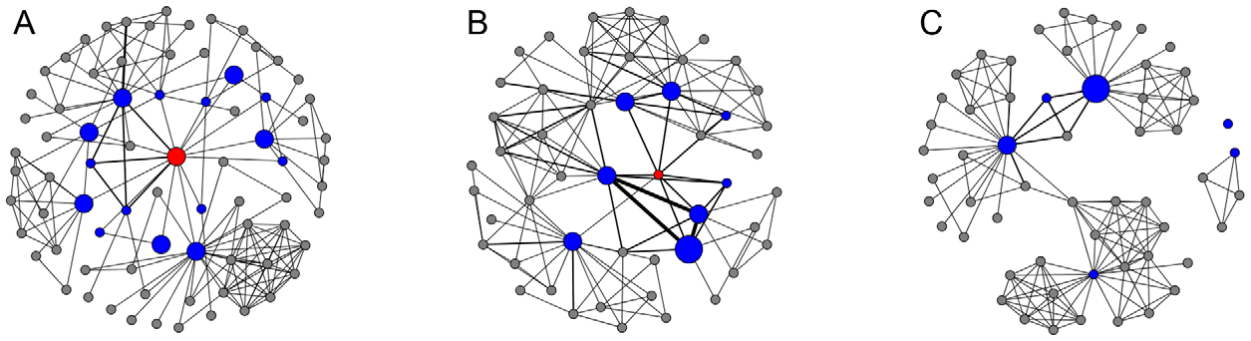
Illustration of egocentric scientific collaboration network evolution. (A) and (B) are the T-2 egocentric scientific collaboration networks of the same eminent scientist in two consecutive non-overlapping time windows (window size = 5 years). The red node is the center of the network, i.e. the superstar. The blue and gray nodes are the first and second tier neighbors of the superstar in that particular time window. The sizes of the nodes and thickness of the edges in the figure are proportional to the numbers of journal articles published by the scientists and the numbers of journal articles co-authored by the pairs of collaborations. (C) is the T'-2 network after the superstar's death. The blue nodes are the dead superstar's first tier neighbors in the last window before his death (the former collaborators). The gray nodes are the neighbors of the former collaborators in the first window after the superstar's death.

Then we define the *academic age* of a scientist as the number of years since his/her first academic publication. Starting from age 0, for every age *y* of a scientist, the egocentric network is constructed using the co-authorship of research articles published from age *y* to *y*+4 (both inclusive, hence forming a window of 5 years). Figs. 3A and B illustrate the egocentric scientific collaboration networks of the same scientist (the red dot) in two consecutive non-overlapping time windows.

Once the egocentric networks of all windows are formed, we measure the scale and connectivity of the networks with four parameters: numbers of nodes *N*, number of edges *M*, clustering coefficient *c* and network efficiency *e*. The clustering coefficient *c* is calculated as follows: 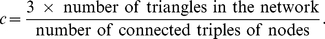
(1)


**Figure 4:**
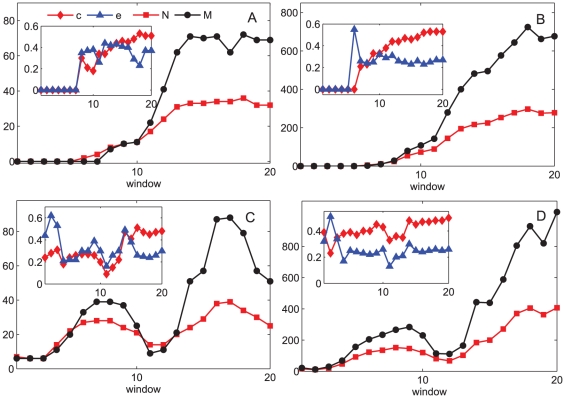
Measures of parameters of two egocentric scientific collaboration networks in their first 20 windows. Figures labeled A–B and C–D represent two scientists respectively. A and C: Numbers of nodes *N*, number of edges *M*, clustering coefficient *c* and network efficiency *e* in T-1 networks. B and D: Numbers of nodes *N*, number of edges *M*, clustering coefficient *c* and network efficiency *e* in T-2 networks in each window. The two scientists have their collaboration networks evolve in two different patterns. The egocentric networks of the first scientist (A and B) have a boost in size during window 10 to 13, while the egocentric networks of the second scientist (C and D) have two peaks of their sizes around window 7 and 16.

The clustering coefficient measures the conditional probability that two scientists may collaborate if they both collaborate with same (third-party) scientist. The network efficiency *e* is obtained by:
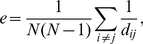
(2) where 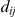 is the shortest path distance between node *i* and *j*. The network efficiency of a fully connected network is 1, whereas for a network of isolated nodes, its efficiency will be 0. Fig. 4 shows the measure of sizes and connectivity of the egocentric collaboration networks of two scientists in the first 20 windows of their academic careers.

Previous research of the US airport network [2] indicates that large complex system can display stationary “macroscopic” structure properties but retain intensive “microscopic” evolution over time. Despite of the ubiquitous global structure of scientific collaboration networks in different fields [13], [14], [18], our analysis shows that same to the airport network, the collaboration network is also intensively evolving at the local scale, where collaborations between scientists are rather temporal than stationary. Furthermore, Fig. 4 also shows that the scientists may have their egocentric collaboration networks evolving in entirely different tracks. The different patterns of evolution can be caused by the fact that in a certain period of time the scientist switches his/her work emphasis to (for example) clinical duties or that at some time the scientist is granted a large amount of money and is able to make more collaborations with peer researchers. Hence we argue that when elaborating a model of the evolving scientific collaboration network and other social networks, except for considering the growing mechanism based on existing topology [14], [18] and modifying connections as a feedback of the dynamical process on the network [8], future study should also take the ability of nodes attracting connections and the life spans of links as intrinsic properties embedded in the systems.

### Targeted attacks on the networks

Recent studies have shown that, following the death of an eminent life scientist (“superstar”), collaborators experience a 5% to 8% decline in their publication rates [19]. Yet, apart from numerical simulations [9], there are few reports regarding the structural response to real life “attacks” on the scientific collaboration networks (or in any other application domain). In this section we evaluate the impact of sudden deaths of superstars to their former collaborators' scientific collaboration networks in order to capture the robustness and resilience of these naturally evolving complex systems.

Twenty one superstars who died unexpectedly are selected as the subject of our study. We define the “former collaborators” of a dead superstar as the superstar's direct collaborators in five years preceding death. To study the impact of the superstars' sudden death, we compare the collaboration networks of the former collaborators in the last 5 years before the superstar's death and in the first 5 years afterwards. The T-1 and T-2 egocentric networks of the dead superstars in the last window characterize, respectively, the collaboration among the former collaborators and their collaboration networks right before the death of the superstar. Then, in the first 5-year window after the superstar's death, two new networks T′-1 and T′-2 are constructed analogously to T-1 and T-2 networks, as shown in Fig. 3C, but with the publications of the former collaborators in this certain period. The before and after-death networks T-1 and T′-1 have almost identical nodes, while for the T-2 and T′-2 networks, the network components can be quite different.

Having constructed the former collaborators' collaboration networks in two consecutive windows, we measure the changes (Δ*N*, Δ*M*, Δ*c*, and Δ*e*) of the number of nodes *N*, number of edges *M*, clustering coefficient *c* (Eqn. 1) and network efficiency *e* (Eqn. 2). Table 1 presents the average values of the parameters of the networks before and after the sudden deaths of the superstars as well as the average change of parameters as a percentage. The results show that in comparison with the T-1 networks, the number of nodes in the T′-1 networks only decreases by about two while the number of edges decreases sharply and along with the disappearance of edges, the clustering coefficient and network efficiency have both dropped significantly. Comparing to the T-2 networks, in the T′-2 networks the number of nodes and number of edges have increased by certain amount while the clustering coefficient varies by a small proportion and the network efficiency of the networks decreases by a half.

**Table 1:** Parameter changes of the former collaborators' networks after superstars' deaths.

	*N*	*M*	*c*	*e*
T-1 (before death)	12.57	28.24	0.48	0.75
T'-1 (after death)	10.33	4.57	0.08	0.11
Change in %	−18%	−84%	−83%	−86%
T-2 (before death)	81.57	203.90	0.48	0.43
T'-2 (after death)	105.29	306.81	0.51	0.21
Change in %	+29%	+50%	+7%	−50%

Average values of network parameters (i.e. number of nodes *N*, number of edges *M*, clustering coefficient *c* and network efficiency *e*) and the changes of these parameters in the collaboration networks of superstars' former collaborators. Assuming a superstar died in year *y*, *T*−1 and *T*−2 networks are his egocentric networks containing only the first tier neighbors and both the first and second tier neighbors from year *y*−4 to *y*. 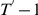 and 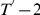 networks are the former collaborators' collaboration networks of themselves and with their first tier collaborators from year *y*+1 to *y*+5. After the superstars' death, the former collaborators tend to disconnect with each other and find other collaborations elsewhere.

This result suggests that the sudden deaths of the superstars have stimulated their former collaborators to rearrange their networks in an efficient manner. To determine whether the impact of sudden death is significantly different from the natural network evolution (i.e. without the sudden death of the superstar), two non-parametric statistical tests are conducted.

**Test 1**: 77 scientists are selected from a group of eminent life scientists as the control group. Having a superstar suddenly died in age *y*, we first find all the scientists in the control group who were still active in research in age *y*+5. Then we measure the properties of T-1 and T-2 networks of control groups scientists in the window of age *y*−4 to *y* and the properties of T'-1 and T'-2 networks in the window of age *y+*1 to *y+*5. For each change of the parameters, say 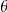, of each suddenly died scientist, we want to know whether it falls into the range of all the 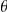
's of the control group. Of the active superstars, let 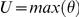 and 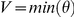. For each of the dead scientists *i*, if 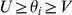, let 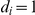 (else 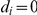). Then the probability of any dead superstars' 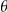 fall inside *U* and *V* is:
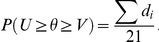



The results of the measured parameters are summarized in Table 2. Almost all the parameter changes of individual egocentric networks after the sudden death of superstars fall in range of the parameter changes due to normal evolution of the collaboration networks.

**Table 2:** Results of Test 1 and Test 2.

	Change between	Δ*N*	Δ*M*	Δ*c*	Δ*e*
Test 1 (*P*)	T'-1 and T-1	21/21	20/21	21/21	21/21
	T'-2 and T-2	21/21	20/21	20/21	21/21
Test 2 (*p*-value)	T'-1 and T-1	0.52	0.45	0.68	0.36
	T'-2 and T-2	0.27	0.24	0.22	0.53

The test results of Test 1 (*P*) and Test 2 (*p*-value) for the changes of numbers of nodes Δ*N*, number of edges Δ*M*, clustering coefficient Δ*c* and network efficiency Δ*e* in 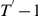 and 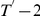 networks comparing to *T*−1 and *T*−2 networks. The large values of *P*'s (close to 1) and *p−value*'s (larger than 0.05) indicate that the deaths of superstars did not have significant impact on the way of evolving of their collaborators.

In **Test 2** the Wilcoxon's two-sided rank sum test is used. The observed data is the parameter changes 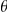 of all the 21 sudden deaths; the control group is the 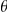 of 42 normal superstars, who are also in the control group in Test 1 and are removed from their egocentric networks at ages following the same academic “age-of-death” distribution as the observed data. For each 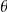, we test the observed data and control group for the null hypothesis: observed and control group data are independent samples from identical continuous distributions with equal medians, against the alternative that they do not have equal medians. The *p*-values for each parameter is presented in Table 2. Let significance level be 95%, then all the changes of network parameters of dead superstars are actually not different (i.e. *p*-values are all larger than 0.05) from the change of network evolutions of active scientists.

Our statistical tests show that there is no evidence that the sudden death of a superstar may have a significant impact on the evolution of its collaborators' scientific collaboration networks. Previous research shows that improving the robustness of diverse networks often involves increasing the redundancy of the network at critical positions [20]. Our findings of the evolving scientific collaboration network reveal, on the other hand, that the network with intensive evolution also show great resilience even under attacks on important nodes, which could severely disrupt the functionality of stationary networks.

## Discussion

Of course, the premature deaths of eminent scientists may be considered a great loss to their particular discipline. Nonetheless, it is known that after the (unanticipated) deaths of some eminent scientists, the scientific productivity of collaborators suffer from a 5% – 8% drop. In this paper we have examined, from another aspect, the impact of the sudden deaths of these superstars to the structure evolution of their former collaborators' collaboration networks. We have firstly shown that the scientific collaboration network is a complex system which intensively evolves at the local level. Most collaborations among scientists have short life spans and the relative incidence of long term collaboration is very low. We have compared the behavior of network evolution between collaborators of suddenly deceased eminent scientists and active ones. Surprisingly, statistics show that the evolution of collaborators' networks are not affected by the sudden deaths of the superstars.

In particular, we have observed that the egocentric scientific collaboration networks evolve in such a manner that: direct collaborators of a superstar in one period of time tends not to collaborate with each other in the next, whereas the collaborators' own egocentric networks grow bigger. This evolution pattern is actually an analogy to the diffusion process on an arbitrary form of network, where nodes can generate a stimulus and spread it out to their first then second tier neighbors and so on. Hence we conjecture that, rather than mapping the social networks of scientists, the scientific collaboration network is actually the “trace” of information propagation on a larger and denser invisible social network than the trace itself.

Actually the trace of information propagating and disease spreading in human society share the same evolution mechanism with scientific collaboration network and that the redundancy of the underlying social structure in fact acts as a protection mechanism when these networks are under attack. From this perspective, future study of effective network attacks (such as immunization strategies) should consider the underlying rapid evolving social structure. Moreover, the designing of robust information transmission systems could also gain from the robust system formed by human social and collaborative endeavors. For example in the Internet, routing strategies with constantly changing paths between nodes might give extra robustness to the system even under targeted attacks.

## Materials and Methods

In this paper the collaborations of three groups of US based life scientists are studied. The first group are the scientists listed in the Faculty Roster of the Association of American Medical Colleges until the end of 2010. The second group contains 77 eminent life scientists (“superstars”), including (i) current members of National Academy of Sciences major in life science; (ii) emeritus members of National Academy of Sciences major in life science; (iii) top 500 highly cited life scientists retrieved from ISI Web of Knowledge until the end of 2010. Moreover all of the 77 scientists had been active in their academic life for not less than 10 years and had collaborated with not less than 20 other scientists in the Faculty Roster. The third group of scientists are 21 life scientists who died unexpectedly and prematurely in the early stage of their scientific career and had comparable academic achievements with the previous group of superstars at the time of their death [19]. These 21 scientists had also been active in their academic life for not less than 10 years and had collaborated with not less than 20 other scientists in the Faculty Roster.

Scientists are connected only when they co-author a journal article. The publication information are retrieved from online database PubMED, which is provided by the National Library of Medicine and stores intact biomedical research literature. The authors' names in PubMED are stored in the form of name identifier which takes the initials of the first names and the whole last name, i.e. Xiao Fan Liu is stored as XF Liu. However in the Faculty Roster which stores the full names of all the faculties, some of the names may have the same identifiers. For example the identifiers of John Doe and Jane Doe are all J Doe. Hence from the information provided by PubMED we cannot determine whether a paper published by J Doe is actually written by John Doe or Jane Doe. In our work, different names with the same identifiers are eliminated from the Faculty Roster, thereby reducing the size of the Faculty Roster to 112,753.

**Figure 5:**
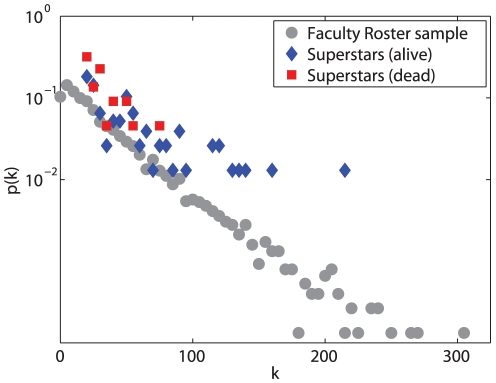
Degree distributions of three groups of scientists. The degree distributions of 7555 samples from the Faculty Roster, 77 eminent life scientists and 21 suddenly died eminent life scientists are shown in the figure. The average degree of the three groups are 31.83, 56.56 and 35.29.

The superstars are not only excellent in their academic achievements but also important in terms of network measure in the network of scientists. Constructing a scientific collaboration network covering all the publications the scientists have in their life time, Fig. 5 shows the degree distribution of the three groups of scientists. The degree distribution of scientists in the Faculty Roster follows an exponential distribution and has an average degree of 31.83; the average degree of the 77 eminent life scientists is 56.56, which is almost twice as much as that of all the scientists in the Roster; and the average degree of the 21 scientists who died suddenly is 35.29. Note that the 21 scientists died in their early ages and had obtained comparable academic achievements with the 77 eminent ones at the time of death, we can assume that their collaboration network would also have continued to grow to comparable sizes as the ones alive.
